# Generalized Linear Models with Covariate Measurement Error and Zero-Inflated Surrogates

**DOI:** 10.3390/math12020309

**Published:** 2024-01-17

**Authors:** Ching-Yun Wang, Jean de Dieu Tapsoba, Catherine Duggan, Anne McTiernan

**Affiliations:** 1 Division of Public Health Sciences, Fred Hutchinson Cancer Center, P.O. Box 19024, Seattle, WA 98109-1024, USA; 2 Vaccine and Infectious Disease Division, Fred Hutchinson Cancer Center, P.O. Box 19024, Seattle, WA 98109-1024, USA

**Keywords:** measurement error, surrogate, zero-inflated data, 62E20, 62F10, 62J12

## Abstract

Epidemiological studies often encounter a challenge due to exposure measurement error when estimating an exposure–disease association. A surrogate variable may be available for the true unobserved exposure variable. However, zero-inflated data are encountered frequently in the surrogate variables. For example, many nutrient or physical activity measures may have a zero value (or a low detectable value) among a group of individuals. In this paper, we investigate regression analysis when the observed surrogates may have zero values among some individuals of the whole study cohort. A naive regression calibration without taking into account a probability mass of the surrogate variable at 0 (or a low detectable value) will be biased. We developed a regression calibration estimator which typically can have smaller biases than the naive regression calibration estimator. We propose an expected estimating equation estimator which is consistent under the zero-inflated surrogate regression model. Extensive simulations show that the proposed estimator performs well in terms of bias correction. These methods are applied to a physical activity intervention study.

## 1. Introduction

In biomedical research, regression analysis is an important tool to understand associations between disease outcomes and risk factors. In practice, however, a risk factor may not be measured precisely. This problem is often called covariate measurement error [1–3]. We consider an example when a biomarker is a risk factor for a disease outcome. In practice, the biomarker may have seasonal, daily, or even hourly variation, and a single measurement is prone to a covariate measurement error from instrumentation or human error. Hence, an average of an infinite number of the biomarker measurements during a specified period of time is, therefore, a more meaningful covariate variable than the average of a few observed measurements. However, in practice it is not feasible to make such measurements, and thus studies often rely on single measures at a specific time point with associated measurement error.

Physical activity and nutrient intake are important risk factors for disease incidence and mortality. However, physical activity and nutrient intake data may be measured with errors since they are generally self-report data. This issue is important since measurement error in diet or physical activity may have an attenuation effect on the regression coefficients of exposures in the range of approximately 20% to 50% [4–6]. That is, an odds ratio of 1.5 from diet or physical activity may be reduced to the range of 1.22 to 1.38 due to measurement errors in these measures. In addition, an important challenge in this research is that some physical activity or dietary data may have a zero value, such as 0 metabolic equivalent (MET) hours per week from moderate or vigorous physical activity or 0 alcohol intake. One MET is defined as the amount of oxygen consumed while at rest per kilogram of body weight [7]. A 3 MET activity expends three times the energy used by the body at rest. Hence, if a person does a 3 MET activity for 4 h in a week, he or she has done 12 MET hours of physical activity in a week. A naive method without taking into account measurement error may lead to biased effect estimation in regression analysis, and the bias is attenuation in most (but not all) cases [8]. A standard bias correction for measurement error without taking into account a subset of individuals with zero exposure value may be biased in the effect estimation.

One motivating example of our methodology research is covariate measurement error associated with the measurement of physical activity in the APPEAL study (A Program Promoting Exercise and Active Lifestyles; APPEAL: Clinicaltrials.gov NCT00668161) [9]. APPEAL was a year long randomized controlled trial of moderate-to-vigorous intensity exercise vs. control (no exercise) among 202 healthy, sedentary adults recruited between 2001 and 2004 primarily through physician practices, and randomized to an exercise program (*n* = 100) or a control group (*n* = 102). The trial was designed to test the effects of exercise on biomarkers of colon cancer and other physiologic and psychosocial outcomes. Numerous case-control and cohort studies have found an inverse association between physical activity and risk of colon cancer [10]. Physical activities are commonly quantified by determining the energy expenditure in kilocalories or by using the MET of the activity. A question of interest is whether there is an association between physical activity via MET-hours/week and c-reactive protein, a biomarker of inflammation, with elevated levels of CRP associated with risk of developing colon cancer. The true average of MET-hours/week is an unobserved variable that is the average of an infinite number of MET-hours/week scores. However, in practice it is not possible to obtain this measure and, thus, the true average of MET-hours/week scores cannot be observed.

In the motivating example given above, two methodology challenges are involved. The first challenge is regression analysis with covariate measurement error, which is due to physical activity (MET-hours/week). The observed error-prone variable is typically called a surrogate variable for the true but unobserved exposure. The second challenge is the zero-inflated surrogate model because some individuals may have zero MET-hours/week. The zero-inflated surrogate issue in some similar research examples is also called truncation of the observed surrogates. In our problem, the second challenge (zero-inflated surrogate modeling) is added to the first challenge (covariate measurement error). Methods for covariate measurement error have been well developed. For example, regression calibration (RC) for covariate measurement error is to replace an error-prone covariate by its conditional expectation given the observed covariates [11]. In linear regression, the RC estimator is a consistent estimator for regression coefficients (Buonaccorsi, 2010, Chapter 5) [12]. However, for logistic and Cox regression, it is known that it is not consistent (Carroll, et al., 2006, Chapter 4) [2]. There is further research on refinement of RC for logistic and Cox regression [13,14]. Another general approximation approach for covariate measurement error is the simulation extrapolation (SIMEX) approach [15,16]. An advantage of SIMEX is that it has the advantage of being easy to implement. There are methods to address the situation when the surrogate variables may be truncated (which is in general the same as zero-inflated surrogate modeling). Tooze et al. investigated a likelihood approach for repeated measures data with clumping at zero [17]. When the observed exposure variables are truncated by a lower limit, the estimation of the disease–exposure association due to measurement error and truncation may not always be attenuation [18].

As discussed above, there is relatively limited research that addresses the issue of measurement error when some individuals may have a zero value (or lower limit) in the observed surrogates. The main objective of the paper is to develop and apply methods to adjust for measurement error in generalized linear models when the observed surrogates may be truncated at a low value (such as 0) among some individuals. The paper is organized as follows: In Section 2, we describe the statistical models for the problem of interest, and discuss the bias issue when we apply a naive RC estimator without taking into account the zero-inflated surrogates. In Section 3, we study a regression calibration estimator for this problem. In Section 4, we propose a maximum likelihood estimator via expected estimating equations for this problem. In Section 5, the results from simulation studies are presented. In Section 6, we apply the methods to the APPEAL study data. We discuss the advantages and limitations of the proposed EEE estimator in Section 7. Concluding remarks are given in Section 8.

## 2. Statistical Models and Naive RC Estimator

We assume that the total sample size of the study cohort is n. The regression model of interest is the generalized linear model. Let $Y_{i}$ be the response variable, $X_{i}$ be the unobserved true covariate (dietary intake or physical activity) that cannot be precisely measured, and $Z_{i}$ be the vector of covariates which is available for all individuals, i,i=1,…,n. For simplicity of presentation, the true unobserved exposure X is assumed to be a scalar throughout this paper. The main interest is to estimate the vector of regression coefficients $\beta \equiv {\left(\beta_{0},\beta_{1},\beta_{2}^{'}\right)}^{'}$ in the following regression model:

(1) $$E\left(Y_{i}|X_{i},Z_{i}\right)=g\left(\beta_{0}+\beta_{1}X_{i}+\beta_{2}^{'}Z_{i}\right),$$

where g(⋅) is a specified function. Model (1) contains many important regression models. For example, g(u)=u in linear regression, while $g(u)={\left(1+e^{-u}\right)}^{-1}$ in logistic regression. The goal of the research is to develop valid estimation methods for the regression coefficients β. For the true unobserved covariate $X_{i}$, we assume that there are $k_{i}$ non-negative surrogate variables $W_{ij},\;j=1,\ldots ,k_{i}$ such that $W_{ij}=max(c,W_{ij}^{*})$, where c is a detection limit, $W_{ij}^{*}=X_{i}+U_{ij}$, in which $U_{ij}$ is an independent measurement error with $E\left(U_{ij}\right)=0$. Let $\eta_{ij}$ be the indicator function for a positive $W_{ij}$ value, that is, $\eta_{ij}=I\left[W_{ij}>c\right]$. In a covariate measurement error problem when the surrogates are not truncated, replicates $W_{ij},j=1,\ldots ,k_{i}$, are used to estimate the measurement error variance where $k_{i}$ is the number of replicates. We use notation ${\tilde{W}}_{i}$ for $(W_{i1},\ldots ,W_{ik_{i}}),{\tilde{W}}_{i}^{*}$ for $\left(W_{i1}^{*},\ldots ,W_{ik_{i}}^{*}\right)$, and ${\tilde{\eta }}_{i}$ for $\left(\eta_{i1},\ldots ,\eta_{ik_{i}}\right)$.

To understand the RC estimator, we consider a special linear regression case that $Y_{i}=\beta_{0}+\beta_{1}X_{i}+e_{i}$, where $e_{i}$ is a mean-zero random residual term. Assume $W_{ij}^{*}=X_{i}+U_{ij},j=1,\ldots ,k$, then it is easily seen that $E(Y_{i}|{\tilde{W}}_{i}^{*})=\beta_{0}+\beta_{1}E\left(X_{i}|{\tilde{W}}_{i}\right)$. From this argument, it is seen that under the special linear regression case above, replacing an unobserved true $X_{i}$ with $E(X_{i}|{\tilde{W}}_{i}^{*})$ will lead to a consistent estimator. This method is often called the RC estimator [2]. In this case, $E(Y_{i}|{\tilde{W}}_{i}^{*})$ is the calibration function. We may also use $E(Y_{i}|{\overset{‾}{W}}_{i}^{*})$, where ${\overset{‾}{W}}_{i}^{*}=\sum_{j=1}^{k}\;W_{ij}^{*}/k$, as the calibration function to replace the unobserved $X_{i}$. If replicates $W_{ij}^{*},j=1,\ldots ,k_{i}$ are from a normal distribution, then $E(Y_{i}|{\overset{‾}{W}}_{i}^{*})=E(Y_{i}|{\tilde{W}}_{i}^{*})$ [14]. Let $\mu_{x}$ and $\sigma_{x}$ denote the mean and standard deviation of any random variable X, respectively. Calculation of the conditional expectation of the unobserved exposure given the surrogates can be obtained based on a bivariate normal assumption such that

$$E(X_{i}|{\overset{‾}{W}}_{i}^{*})=\mu_{x}+\sigma_{x}^{2}{\left(\sigma_{x}^{2}+\sigma_{u}^{2}/k\right)}^{-1}({\overset{‾}{W}}_{i}^{*}-\mu_{x}).$$

Therefore, $E(Y_{i}|{\overset{‾}{W}}_{i}^{*})=\beta_{0}+\beta_{1}^{*}{\overset{‾}{W}}_{i}^{*}$, then $\beta_{1}^{*}=\{\sigma_{x}^{2}{\left(\sigma_{x}^{2}+\sigma_{u}^{2}/k\right)}^{-1}\}\beta_{1}$. From this calculation, a naive estimator using ${\overset{‾}{W}}_{i}^{*}$ as a replacement for $X_{i}$ will have an attenuation effect. When Z is in the model, a standard RC estimator is to replace $X_{i}$ with $E(X_{i}|{\overset{‾}{W}}_{i}^{*},Z_{i})$. This can be done by a multivariate-normal assumption with a conditional mean formula similar to the formula given above. However, a more practical approach is via a semiparametric RC approach by assuming a working regression model of $E(W_{ij}^{*}|W_{ij^{'}}^{*}Z_{i})=\alpha_{0}+\alpha_{1}W_{ij^{'}}^{*}+\alpha_{2}^{'}Z_{i}$, where $j\neq j^{'}=1,\ldots ,k$, and ${\left(\alpha_{0},\alpha_{1},\alpha_{2}^{'}\right)}^{'}$ is the vector of regression coefficients. This semiparametric RC estimator does not assume a multivariate normality assumption of the observed surrogates and covariates [19,20].

However, in our problem, the observed $W_{ij}$ is different from $W_{ij}^{*}$ if $W_{ij}^{*}<c$. Using $W_{ij}$ data will likely overestimate $\mu_{x}$, but underestimate $\sigma_{x}$, and $\sigma_{u}$ since $W_{ij}=c$ if $W_{ij}^{*}<c$. For linear regression with truncated surrogates, standard RC may be biased because $E\left(X_{i}|{\overset{‾}{W}}_{i}\right)$ will be different from $E(X_{i}|{\overset{‾}{W}}_{i}^{*})$. One naive approach is to use the observed $W_{ij}$ as $W_{ij}^{*}$, without taking into consideration the truncated surrogates, to calculate the RC estimator. We call this estimator a naive RC (NRC) estimator. As discussed above, the NRC estimator is biased even when the main regression model is linear. The asymptotic variance of the NRC estimator can be obtained by a sandwich variance estimator where the vector of the estimating equations is obtained by stacking the estimating equations for β and the nuisance parameters involved in the calculation of the calibration function $E(X_{i}|{\tilde{W}}_{i}^{*},Z_{i})$ (but noting that the NRC estimator assumes ${\tilde{W}}_{i}$ is the same as ${\tilde{W}}_{i}^{*}$). However, if there are many covariates in the modeling of the calibration function, then it will be computationally easier to use bootstrap variance estimation to obtain the standard errors.

## 3. Regression Calibration for Zero-Inflated Surrogates

The NRC estimator described in the previous section does not take into account zero values due to truncation. Now, we consider calibration based on truncate surrogates due to zero values. To understand the method, we first consider a linear regression model $Y_{i}=\beta_{0}+\beta_{1}X_{i}+\beta_{2}^{'}Z_{i}+e_{i}$, where $e_{i}$ has mean 0, and is independent of $X_{i}$ and $Z_{i}$. Then, $E\left(Y_{i}|{\tilde{W}}_{i},Z_{i}\right)=\beta_{0}+\beta_{1}E\left(X_{i}|{\tilde{W}}_{i},Z_{i}\right)+\beta_{2}^{'}Z_{i}$. That is, replacing $X_{i}$ with $E\left(X_{i}|{\tilde{W}}_{i},Z_{i}\right)$ in the regression analysis may be a valid approach. Let ${\hat{X}}_{i}\equiv E(X|\tilde{W},Z)$. The estimating equation for the RC estimator can be expressed as

(2) $$\sum_{i=1}^{n}{\left(1,{\hat{X}}_{i},Z_{i}^{'}\right)}^{'}\{Y_{i}-(\beta_{0}+\beta_{1}{\hat{X}}_{i}+\beta_{2}^{'}Z_{i})\}=0.$$

Hence, when $Y_{i}$ given $\left(X_{i},Z_{i}\right)$ is linear, we have the following result:

### Proposition 1.

*Assume the surrogate variables* $W_{ij}^{*},j=1,\ldots ,k_{i}$ *may be truncated by a lower limit, and the truncation indicator* ${\tilde{\eta }}_{i}$ *is independent of* $Y_{i}$ *given* $\left(X_{i},Z_{i}\right)$. *If* $Y_{i}=\beta_{0}+\beta_{1}X_{i}+\beta_{2}^{'}Z_{i}+e_{i}$, *where* $e_{i}$ *has mean 0, and is independent of* $X_{i}$ *and* $Z_{i}$. *Then the RC estimator solving* (2) *is a consistent estimator of* β.

The proof of Proposition 1 is given in Appendix A. We note that because of the surrogate assumption, the measurement errors $U_{ij}$ and $e_{i}$ are independent, which is needed to ensure that estimating Equation (2) is unbiased. Hence, for linear regression with zero-inflated surrogates, the RC estimator is consistent. However, when the mean function of $Y_{i}$ given $X_{i},Z_{i}$ is not linear, the RC estimator may be biased since the expectation of the estimating score will no longer be zero. For logistic regression, $pr\left(Y_{i}=1|X_{i},Z_{i}\right)=H\left(\beta_{0}+\beta_{1}X_{i}+\beta_{2}^{'}Z_{i}\right)$, where $H(u)=\{1+exp(-u){\}}^{-1}$ is the logistic function. Although the RC estimator is not consistent, the RC estimator can be considered as an improved estimator of the NRC estimator described in the last section. The calibration function can be calculated based on the likelihood function. We use notation ℒ(X) to denote a likelihood function for any random variable X, and ℒ(Y∣X) to denote a conditional likelihood function of Y given X, for any two random variables X and Y. Generally, the conditional calibration function can be calculated by the following:

(3) $$E\{X_{i}|{\tilde{W}}_{i},Z_{i}\}=\frac{\int_{x}\;x\prod_{j}\;{\left\{ℒ\left(W_{ij}|X_{i}\;=\;x,Z_{i}\right)\right\}}^{\eta_{ij}}{\left\{ℒ\left(W_{ij}\;=\;c|X_{i}\;=\;x,Z_{i}\right)\right\}}^{1-\eta_{ij}}ℒ\left(Z_{i},X_{i}\;=\;x\right)dx}{\int_{x}\;\prod_{j}\;{\left\{ℒ\left(W_{ij}|X_{i}\;=\;x,Z_{i}\right)\right\}}^{\eta_{ij}}{\left\{ℒ\left(W_{ij}\;=\;c|X_{i}\;=\;x,Z_{i}\right)\right\}}^{1-\eta_{ij}}ℒ\left(Z_{i},X_{i}\;=\;x\right)dx}.$$

In (3), we note that $ℒ\left(W_{ij}=c|X_{i}=x,Z_{i}\right)=ℒ\left(U_{ij}\leq c-x\right)$. From the argument given above, the RC estimator can be obtained by replacing an unobserved $X_{i}$ by $E\left\{X_{i}|{\tilde{W}}_{i},Z_{i}\right\}$ based on (3). The asymptotic variance of the RC estimator can be obtained by a stacked sandwich estimator that is similar to the one for the NRC estimator described in the last section, or by bootstrap variance estimation.

## 4. Expected Estimating Equation Estimator

We now develop another approach to this problem via the maximum likelihood (ML) estimation. We first take a different viewpoint linking the ML estimation and the conditional expectation of the *full data estimating equation*, namely, the estimating equation when there is no measurement error. The full data likelihood, $ℒ\left(Y_{i}|X_{i},Z_{i}\right)$, is the likelihood function of $Y_{i}$ given $\left(X_{i},Z_{i}\right)$. The full data estimating equation for β can be expressed as $\sum_{i=1}^{n}\;\phi \left(Y_{i},X_{i},Z_{i},\beta \right)=0$, in which $\phi \left(Y_{i},X_{i},Z_{i},\beta \right)$ is the derivative of $log\left\{ℒ\left(Y_{i}|X_{i},Z_{i}\right)\right\}$ with respect to β. Because the true $X_{i}$ is not observed, the full data estimating equation can not be directly applied to the data. With the observed data, the estimating score will be from the likelihood of $Y_{i}$ given $Z_{i}$ and $W_{i}$, denoted by $ℒ\left(Y_{i}|Z_{i},W_{i}\right)$. If the distribution of $\left({\tilde{W}}_{i},X_{i},Z_{i}\right)$ does not involve β, then

$$\begin{array}{r} \frac{\partial }{\partial \beta }logℒ\left(Y_{i}|{\tilde{W}}_{i},Z_{i}\right)=\frac{(\partial /\partial \beta )\int_{x}\;ℒ\left(Y_{i}|X_{i},Z_{i}\right)ℒ\left({\tilde{W}}_{i}|X_{i}=x,Z_{i}\right)ℒ\left(X_{i}=x,Z_{i}\right)dx}{ℒ\left(Y_{i},{\tilde{W}}_{i},Z_{i}\right)}\\ =E\{\left.\frac{\partial }{\partial \beta }logℒ\left(Y_{i}|X_{i}=x,Z_{i}\right)\right|\;Y_{i},{\tilde{W}}_{i},Z_{i}\}. \end{array}$$

From the equations given above, the likelihood-based score of the observed data can be obtained by the conditional expectation of the likelihood-based score of the full data given the observed data. That is, the estimating score for an individual can be expressed as $E\left\{\phi \left(Y_{i},X_{i},Z_{i},\beta \right)|Y_{i},{\tilde{W}}_{i},Z_{i}\right\}$, which is the observed data estimating score. The ML estimator can be obtained from the idea of expected estimating equations [21]. Therefore, the ML estimator can be obtained by solving

(4) $$\sum_{i=1}^{n}E\left\{\phi \left(Y_{i},X_{i},Z_{i},\beta \right)|Y_{i},{\tilde{W}}_{i},Z_{i}\right\}=0.$$

In general, $\phi \left(Y_{i},X_{i},Z_{i},\beta \right)$ does not need to be the full data likelihood-based estimating score. It can be any estimating equation that satisfies $E\left\{\phi \left(Y_{i},X_{i},Z_{i},\beta \right)\right\}=0$. For example, it can be a weighted estimating equation of the ML estimator. The estimator solving (4) is the expected estimating equation (EEE) estimator for β. Let Equation (4) be denoted by S(β,X,Z)=0. Let the EEE estimator be denoted by ${\hat{\beta }}_{\mathrm{eee}}$. The asymptotic distribution of ${\hat{\beta }}_{\mathrm{eee}}$ can be presented as the following result:

### Proposition 2.

*Assume* $Y_{i}$ *given* $\left(X_{i},Z_{i}\right)$ *follows* (1), *and the surrogate variables* $W_{ij}^{*},j=1,\ldots ,k_{i}$ *may be truncated by a lower limit, and the truncation indicator* ${\tilde{\eta }}_{i}$ is *conditionally independent of* $Y_{i}$ *given* $\left(X_{i},Z_{i}\right)$. *Assume* $\phi \left(Y_{i},X_{i},Z_{i},\beta \right)$ *is any estimating equation that satisfies* $E\left\{\phi \left(Y_{i},X_{i},Z_{i},\beta \right)\right\}=0$. *The EEE estimator solving* (4) *is consistent for* β. *Furthermore*, $n^{1/2}({\hat{\beta }}_{\mathrm{eee}}-\beta )$ *is asymptotically normal with mean 0 and asymptotic variance given in* Appendix A.

The proof of Proposition 2 is given in Appendix A. The EEE in (4) can be calculated by the following:

$$\begin{array}{ll} & E\left\{\phi \left(Y_{i},X_{i},Z_{i},\beta \right)|Y_{i},{\tilde{W}}_{i},Z_{i}\right\}\\ & \;=\frac{\int_{x}\;\phi \left(Y_{i},X_{i},Z_{i}\right)ℒ\left(Y_{i}|X_{i}\;=\;x,Z_{i}\right)\left\{\prod_{j=1}^{k_{i}}\;ℒ\left(W_{ij}|X_{i}\;=\;x,Z_{i}\right)\right\}ℒ\left(Z_{i},X_{i}\;=\;x\right)dx}{\int_{x}\;ℒ\left(Y_{i}|X_{i}\;=\;x,Z_{i}\right)\left\{\prod_{j=1}^{k_{i}}\;ℒ\left(W_{ij}|X_{i}\;=\;x,Z_{i}\right)\right\}ℒ\left(Z_{i},X_{i}\;=\;x\right)dx}, \end{array}$$

where $ℒ\left(W_{ij}|X_{i}=x,Z_{i}\right)={\left\{ℒ\left(W_{ij}|X_{i}=x,Z_{i}\right)\right\}}^{\eta_{ij}}{\left\{ℒ\left(W_{ij}=c|X_{i}=x,Z_{i}\right)\right\}}^{1-\eta_{ij}}$. The asymptotic variance of the EEE estimator solving (4) for β can be obtained by a sandwich variance estimator. The vector of the estimating equations is obtained by stacking two sets of estimating equations. The first set is the estimating equations for β and the second set is the nuisance parameters involved in the conditional distribution of $Y_{i}$ given $\left(Z_{i},{\tilde{W}}_{i}\right)$. However, bootstrap variance estimation is another approach to obtain the standard errors of the EEE estimator.

## 5. Simulation Study

We conducted a simulation study to examine the finite sample performance of the NRC, RC, and EEE estimators with the naive estimator that used ${\overset{‾}{W}}_{i}$ for $X_{i}$. In Table 1, we illustrate the situation when the regression model is linear and the observed surrogates may have a zero value among some individuals. That is, the observed surrogates were truncated at c=0 in the simulations. In this table, each individual’s true covariate is $X_{i}$. We first generated $X_{i},i=1,\ldots ,n$, from a normal distribution, where the sample size was n=500, and n=1000, respectively. We generated two replicates $W_{i1}^{*}$ and $W_{i2}^{*}$ for the unobserved $X_{i}$. With $\mu_{x}=1.5,\sigma_{x}=1$, and $\sigma_{u}=0.707$. The percent of non-zero $W_{ij}$ was $\overset{‾}{\eta }=89\%$; 11% of $W_{ij}$ was truncated at 0. We also considered the situation when $\sigma_{u}=1,\;1.5$, and $\sqrt{3}$, respectively, in which the percent of non-zero covariates were $\overset{‾}{\eta }=86\%,\;80\%$, and 77%, respectively. The outcomes were generated based on linear regression with coefficients $\beta_{0}=0.5$ and $\beta_{1}=1$, and the residuals were from a standard normal distribution. In Tables 1–4, “bias” was obtained from the average of the biases of the regression coefficients estimates of the 500 simulation replicates, “SD” was the sample standard deviation of the estimates, and “ASE” was the average of the estimated standard errors of the estimates. The 95% confidence interval coverage probabilities (CP) were also obtained. The standard errors of the estimates were obtained from sandwich variance estimation. From the result of Table 1, the NRC estimator was not much better than the naive estimator. The reason for limited improvement from the NRC over the naive estimator was because of truncated W values. The RC and EEE estimators were consistent with limited biases under this setting, and hence, they were better than the naive and NRC estimators. Under this setting, the RC and EEE were very comparable.

We considered non-normal X in Table 2 to investigate if the estimators were sensitive to the normality assumption in the calculation. We also examined the sensitivity of the estimators to misspecification of the measurement error distribution. On the upper portion of Table 2, the unobserved X was generated from a mixture of two normal distributions; one with mean 2.5 and variance 1, and the other with mean 1 and variance 0.25, and the mixture percentages were (1/3,2/3). The result from the upper portion of the table was similar to that of Table 1, except that there were small biases from the RC and EEE estimators. We found that the RC and EEE showed small biases when the unobserved exposure had a skewed distribution, but the bias was not too large in general. Nevertheless, the RC and EEE estimators were still better than the NRC and naive estimators under this situation. On the lower portion of Table 2, we considered the situation when X was normal but measurement error was from a location/scale-transformed chi-squared distribution and a mixture of two normal distributions, respectively. The specification of the mixture of two normal distributions was the same as the mixture of normal distributions given above. The location/scale-transformed chi-squared distribution has mean 0 and variance $\sigma_{u}^{2}$ after a chi-squared random variable was location/scale-transformed. From the sensitivity analysis, the RC and EEE estimators were not sensitive to mild violation due to a mixture of normal distributions since the biases were considered small. However, the biases may be sensitive to violation of the normality assumption while the true distribution was very skewed, as for chi-squared distributions. The biases were moderate, rather than small, when the errors were from chi-squared distributions.

In Table 3, the data were generated similarly to those in Table 1 but the main model was logistic regression such that $pr\left(Y_{i}=1|X_{i}\right)=H\left(\beta_{0}+\beta_{1}X_{i}\right)$, where the regression coefficients were β=(0,ln(2)) and β=(0,ln(3)), respectively. The findings were similar to those from Table 1 for the situation when β=(0,ln(2)). The biases of the RC and EEE estimators were very small. Although RC is not consistent, it may have limited biases if the relative risk parameter is small to moderate, such as $\beta_{1}=ln(1.5)$ or $\beta_{1}=ln(2)$ when the exposure’s standard deviation is about 1. However, when $\beta_{1}=ln(3)$, the biases of the RC estimator were larger than those of the EEE estimator. The reason is that the RC estimator’s bias will increase if the relative risk parameter is large. The findings are typically similar to those for measurement error in longitudinal data and survival analysis with covariate measurement error [20,21].

In Table 4, we investigated the situation when both X and Z were included in a linear regression model. We first generated $X_{i},i=1,\ldots ,n$ and two replicates $W_{i1}$ and $W_{i2}$ in the same way as those in Table 1. Covariate $Z_{i},i=1,\ldots ,n$, were generated via $Z_{i}=\rho X_{i}/\sigma_{x}+\sqrt{1-\rho^{2}}V_{i}/\sigma_{z}$, where $V_{i}$ were from $N\left(0,\sigma_{z}^{2}\right)$ and independent from $X_{i},\;\sigma_{z}^{2}=1$ and ρ=0.2. The outcomes were generated via $Y_{i}=\beta_{0}+\beta_{1}X_{i}+\beta_{2}Z_{i}+e_{i}$, where $\beta_{0}=0.5,\;\beta_{1}=1$ and $\beta_{2}=-1$, The residuals $e_{i},\;i=1,\ldots ,n$, were generated from a standard normal random variable which was independent of $X_{i}$ and $Z_{i}$. The findings were mostly similar to those from Table 1. That is, the naive and NRC estimators had large biases while the RC and EEE estimators were consistent with limited biases.

## 6. Analysis of APPEAL Data

The design of the APPEAL study was briefly reviewed in the Introduction. In this section, we are interested in investigating the association between physical activity measured via MET hours per week and CRP. The outcome variable of interest is the CRP value at baseline. In the APPEAL study, MET hours per week and other data including biomarkers were collected at both baseline and 12 months (end of study). In the control group who did not receive the exercise intervention, physical activity levels did not change significantly between baseline and 12 months. Hence, it seems reasonable to assume that the two MET-hours/week scores at baseline and 12 months in the control group (n=102) can be treated as replicates. The MET-hours/week data for the exercise intervention group at 12 months were not included in the analysis as the MET-hours/week value changed significantly for study participants randomized to the exercise intervention between baseline and 12 months. As such, these values cannot be treated as replicates. The MET-hours/week scores at baseline and 12 months are surrogate variables (replicates, control arm only) for an unobserved true MET-hours/week score of an individual (unobserved underlying average of a period of time). The true unobserved average MET-hours/week variable is a variable to measure the actual physical activity which cannot be observed. In addition to MET-hours/week, age at baseline was another covariate in the regression analysis.

**Figure 1.**
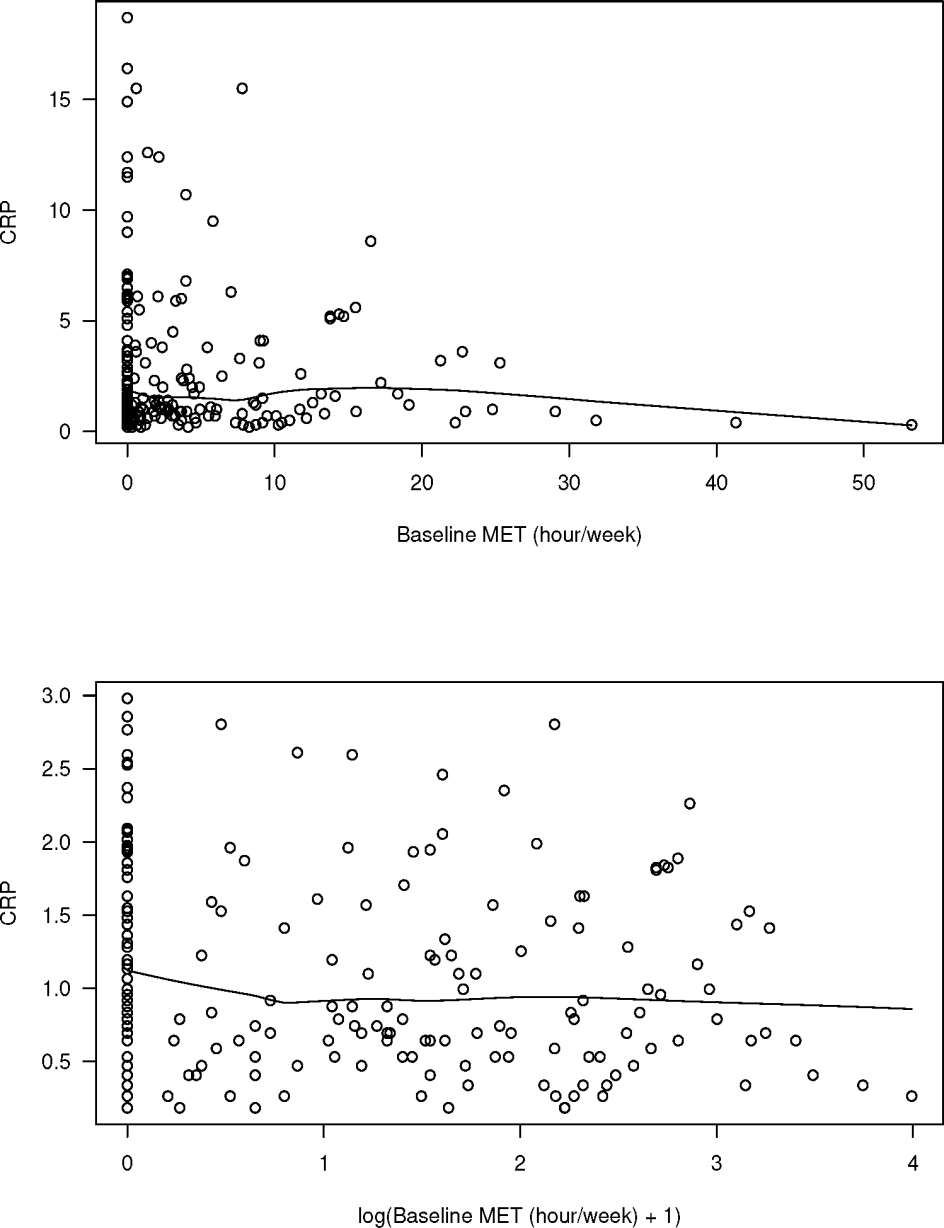
**Upper:** CRP versus MET; **Lower:** log(CRP) versus log(MET+1). The lines were obtained from fitting lowess smoothers.

We first investigated an association between MET-hours/week and CRP at baseline. A scatterplot and a fitted kernel smoother of MET-hours/week and CRP at baseline are shown in the upper portion of Figure 1. The lower portion of Figure 1 is the scatterplot and a fitted kernel smoother of log(MET+1) and log(CRP) at baseline. We excluded 26 individuals with missing data and outliers (defined as values larger than median + 3× interquartile range) for CRP. Hence, a total of 176 individuals are included in the data analysis. The percentage of non-zero log(MET+1) at baseline is 67%, and 68% at 12 month. In our regression analysis, we used the log-transformed data since the transformed data were less skewed.

In this section, the data analysis involved applying our methods to the regression association for the effects of physical activity (MET-hours/week) and age on CRP. The data application here is primarily for the purpose of a demonstration of our new methods. The regression coefficients were estimated based on the naive, RC, CRC, and EEE estimators. The results are given in Table 5. All the four estimators showed that MET was negatively associated with the inflammatory marker CRP; but not significant.

From the naive estimator, when the log(MET+1) score increased by 1 h/week, the CRP, on average, decreased by about 0.07 mg/L. From the NRC, RC, and EEE estimates, when the log(MET+1) score increased by 1 h/week, the CRP, on average, decreased by about 0.1 mg/L. It was observed that the standard errors from the NRC, RC, and EEE estimates were larger than those from the naive estimates. This was a general phenomenon of a bias-efficiency trade-off that has been reported in the measurement error literature, and is consistent with the findings from our simulations. Furthermore, all the four estimates demonstrated a significant effect of age on CRP. On average, an increase of 10 years in age was associated with an increase of approximately 0.15 mg/L in log(CRP).

## 7. Discussion

In the paper, we propose an EEE estimator for generalized linear models with covariate measurement error when the surrogate variables may have zero values among a subset of individuals. Our work is applicable to the situation for more applications when an exposure may be truncated. Our numerical studies show that RC is better than the naive estimator and NRC estimator in general, but it may be biased under some situations. Overall, the EEE estimator has smaller biases. There is a trade-off between bias and efficiency. The EEE has a larger SE due to this. One limitation of the proposed EEE estimator is that it may be biased if the likelihood function of the exposure variable is misspecified. Our simulation results demonstrate that the biases are moderate if the exposure distribution is not too skewed. Future research is needed to develop a non-parametric approach that does not require the exposure variable distribution [22].

In addition to physical activity or dietary data, biomarker measurements are important for the early detection and monitoring of disease progression. Our methods developed in this paper can be applied to biomarker data. When a biomarker is truncated due to a detection limit, decisions are required concerning how to handle values at or below the threshold in order to avoid biasing the parameter estimates. However, biomarkers are often measured with errors for many reasons, such as imperfect laboratory conditions, analytic variability of the assay, or temporal variability within individuals. The statistical modeling of zero-inflated surrogates in this paper can be applied to the situation when biomarker data are truncated due to a detection limit. Further research is needed if longitudinal biomarker, physical activity, or dietary data, are available over time [23–25].

## 8. Conclusions

We have developed an EEE approach for regression analysis with covariate measurement error when the surrogates may be truncated. One limitation of our proposed EEE estimator is that it is not consistent if the covariate distribution or the measurement error distribution is misspecified. In our simulations, the covariates and measurement errors are from normal distributions. Our simulation results demonstrate that if the misspecification is not too extreme, then the bias is typically small. Hence, if the covariates are skewed, then an appropriate (such as a logarithmic) transformation of the data may reduce the skewness of the data. Then the proposed EEE estimator may work well with likely minimal biases.

## Figures and Tables

**Table 1.** Simulation study for linear regression with truncated surrogates.

		Naive	NRC	RC	EEE	Naive	NRC	RC	EEE
	
		*n* = 500				*n* = 1000			

*μ_x_* = 1.5, *σ_x_* = 1, *σ_u_* = 0.707, $\overset{¯}{\eta }$ = 89%									
	
*β*_0_ = 0.5	Bias	0.134	−0.230	−0.002	0.003	0.133	−0.228	−0.003	0.002
	SD	0.093	0.117	0.103	0.103	0.064	0.080	0.072	0.071
	ASE	0.093	0.117	0.106	0.106	0.066	0.083	0.075	0.074
	CP	0.684	0.486	0.972	0.962	0.460	0.180	0.954	0.966
*β*_1_ = 1	Bias	−0.126	0.107	0.004	0.000	−0.127	0.103	0.001	−0.002
	SD	0.050	0.068	0.060	0.060	0.035	0.047	0.043	0.042
	ASE	0.049	0.068	0.061	0.061	0.035	0.048	0.043	0.043
	CP	0.270	0.658	0.958	0.954	0.056	0.446	0.956	0.960

*μ_x_* = 1.5, *σ_x_* = 1, *μ_u_* = 1, $\overset{¯}{\eta }$ = 86%									
	
*β*_0_ = 0.5	Bias	0.301	−0.349	−0.007	−0.006	0.299	−0.343	−0.005	−0.004
	SD	0.096	0.161	0.133	0.132	0.067	0.109	0.091	0.091
	ASE	0.095	0.162	0.136	0.136	0.068	0.113	0.095	0.095
	CP	0.122	0.404	0.960	0.952	0.002	0.106	0.966	0.962
*β*_1_ = 1	Bias	−0.252	0.154	0.006	0.006	−0.252	0.147	0.003	0.002
	SD	0.050	0.096	0.080	0.079	0.035	0.066	0.056	0.056
	ASE	0.049	0.096	0.082	0.082	0.035	0.067	0.057	0.057
	CP	0.002	0.674	0.952	0.958	0.000	0.424	0.948	0.958

*μ_x_* = 1.5, *σ_x_* = 1, *σ*_u_ = 1.5, $\overset{¯}{\eta }$ = 80%									
	
*β*_0_ = 0.5	Bias	0.556	−0.652	−0.035	0.033	0.558	−0.616	−0.018	−0.019
	SD	0.101	0.341	0.244	0.241	0.070	0.217	0.156	0.157
	ASE	0.098	0.325	0.230	0.229	0.069	0.220	0.157	0.158
	CP	0.000	0.462	0.962	0.942	0.000	0.104	0.960	0.960
*β*_1_ = 1	Bias	−0.445	0.263	0.023	0.022	−0.447	0.241	0.011	0.012
	SD	0.048	0.197	0.152	0.150	0.033	0.126	0.097	0.099
	ASE	0.047	0.188	0.144	0.144	0.033	0.128	0.099	0.099
	CP	0.000	0.846	0.960	0.942	0.000	0.558	0.952	0.954

*μ_x_* = 1.5, *σ_x_* = 1, *σ_u_* = $\sqrt{3}$, $\overset{¯}{\eta }$ = 77%									
	
*β*_0_ = 0.5	Bias	0.655	−0.839	−0.057	−0.051	0.657	−0.769	−0.024	−0.025
	SD	0.101	0.609	0.323	0.307	0.070	0.302	0.197	0.198
	ASE	0.098	0.466	0.300	0.296	0.069	0.302	0.198	0.229
	CP	0.000	0.634	0.956	0.922	0.000	0.150	0.956	0.950
*β*_1_ = 1	Bias	−0.519	0.327	0.038	0.034	−0.522	0.287	0.015	0.015
	SD	0.046	0.286	0.204	0.195	0.033	0.170	0.126	0.127
	ASE	0.045	0.263	0.191	0.189	0.032	0.170	0.126	0.148
	CP	0.000	0.972	0.956	0.918	0.000	0.716	0.948	0.930

NOTE: Naive is an estimator that uses the average of two replicates as the covariate, NRC is the naive RC estimator described in Section 2, RC is the RC estimator that uses $\text{E}(X|\tilde{W})$ as the covariate, and EEE is the expected estimating equation estimator described in Section 4.

**Table 2.** Simulation study for linear regression with truncated surrogates; misspecified distribution for covariate *X* or measurement error.

		Naive	NRC	RC	EEE	Naive	NRC	RC	EEE
	
		*n* = 500				*n* = 1000			

X is from a mixture of two normal distributions and the error is normal									

*μ_x_* = 1.5, *σ_x_* = 1, *σ_u_* = 0.707, $\overset{¯}{\eta }$ = 91%									
	
*β*_0_ = 0.5	Bias	0.209	−0.096	0.041	0.036	0.204	−0.101	0.037	0.032
	SD	0.081	0.099	0.097	0.097	0.061	0.074	0.073	0.073
	ASE	0.084	0.105	0.103	0.103	0.060	0.074	0.072	0.073
	CP	0.300	0.878	0.940	0.946	0.074	0.720	0.900	0.916
*β*_1_ = 1	Bias	−0.160	0.038	−0.020	−0.018	−0.158	0.041	−0.018	−0.016
	SD	0.045	0.058	0.057	0.057	0.033	0.043	0.042	0.042
	ASE	0.046	0.061	0.059	0.060	0.032	0.043	0.042	0.042
	CP	0.060	0.920	0.946	0.950	0.002	0.848	0.928	0.928

*μ_x_* = 1.5, *σ_x_* = 1, *σ_u_* = 1, $\overset{¯}{\eta }$ = 86%									
	
*β*_0_ = 0.5	Bias	0.341	−0.199	0.051	0.036	0.336	−0.204	0.050	0.034
	SD	0.084	0.132	0.123	0.125	0.063	0.098	0.090	0.091
	ASE	0.086	0.139	0.130	0.131	0.061	0.098	0.091	0.092
	CP	0.024	0.734	0.928	0.946	0.000	0.460	0.902	0.920
*β*_1_ = 1	Bias	−0.268	0.074	−0.024	−0.017	−0.265	0.076	−0.024	−0.017
	SD	0.045	0.078	0.075	0.076	0.033	0.058	0.054	0.055
	ASE	0.046	0.082	0.078	0.079	0.033	0.058	0.055	0.055
	CP	0.000	0.892	0.938	0.950	0.000	0.744	0.916	0.932

X is normal and the error is from a modified chi-square distribution									

*μ_x_* = 1.5, *σ_x_* = 1, *σ_u_* = 1, $\overset{¯}{\eta }$ = 87%									
	
*β*_0_ = 0.5	Bias	0.384	−0.278	0.082	0.088	0.385	−0.275	0.085	0.091
	SD	0.095	0.169	0.134	0.134	0.067	0.118	0.094	0.094
	ASE	0.093	0.163	0.129	0.129	0.066	0.115	0.091	0.091
	CP	0.012	0.614	0.870	0.850	0.000	0.322	0.816	0.792
*β*_1_ = 1	Bias	−0.295	0.125	−0.038	−0.040	−0.293	0.125	−0.038	−0.040
	SD	0.052	0.101	0.081	0.081	0.036	0.070	0.056	0.056
	ASE	0.050	0.097	0.078	0.078	0.036	0.069	0.055	0.055
	CP	0.000	0.764	0.898	0.890	0.000	0.594	0.880	0.882

X is normal and the error is from a mixture of two normal distribution									

*μ_x_* = 1.5, *σ_x_* = 1, *σ_u_* = 1, $\overset{¯}{\eta }$ = 84%									
	
*β*_0_ = 0.5	Bias	0.376	−0.431	0.024	−0.024	0.380	−0.418	−0.018	−0.018
	SD	0.096	0.196	0.162	0.162	0.069	0.136	0.107	0.107
	ASE	0.096	0.198	0.160	0.161	0.068	0.139	0.112	0.112
	CP	0.030	0.402	0.954	0.958	0.000	0.114	0.954	0.958
*β*_1_ = 1	Bias	−0.311	0.183	0.013	0.013	−0.314	0.175	0.009	0.009
	SD	0.048	0.116	0.098	0.098	0.033	0.080	0.066	0.066
	ASE	0.049	0.118	0.098	0.099	0.035	0.082	0.068	0.068
	CP	0.000	0.724	0.950	0.950	0.000	0.430	0.954	0.956

NOTE: See the footnote of Table 1 for notation.

**Table 3.** Simulation study for logistic regression with truncated surrogates.

		Naive	NRC	RC	EEE	Naive	NRC	RC	EEE
	
		*n* = 500				*n* = 1000			

*μ_x_* = 1.5, *σ_x_* = 1, *σ_u_* = 0.707, $\overset{¯}{\eta }$ = 89%									
	
*β*_0_ = 0	Bias	0.065	−0.190	−0.010	−0.010	0.063	−0.190	−0.012	−0.012
	SD	0.191	0.234	0.203	0.208	0.136	0.169	0.147	0.150
	ASE	0.181	0.224	0.193	0.199	0.128	0.158	0.136	0.140
	CP	0.922	0.836	0.938	0.944	0.892	0.766	0.936	0.942
*β*_1_ = ln(2)	Bias	−0.080	0.083	−0.008	0.007	−0.079	0.083	−0.006	0.008
	SD	0.122	0.154	0.133	0.142	0.085	0.109	0.094	0.100
	ASE	0.115	0.147	0.126	0.134	0.082	0.104	0.089	0.095
	CP	0.868	0.914	0.928	0.930	0.788	0.874	0.936	0.944

*β*_0_ = 0	Bias	0.069	−0.340	−0.014	−0.013	0.065	−0.341	−0.018	−0.016
	SD	0.207	0.266	0.219	0.232	0.148	0.189	0.159	0.169
	ASE	0.197	0.254	0.210	0.223	0.139	0.179	0.148	0.156
	CP	0.930	0.706	0.950	0.948	0.900	0.518	0.928	0.928
*β*_1_ = ln(3)	Bias	−0.116	0.146	−0.035	0.015	−0.114	0.145	−0.034	0.014
	SD	0.159	0.205	0.165	0.190	0.111	0.141	0.115	0.132
	ASE	0.149	0.191	0.155	0.178	0.106	0.135	0.109	0.125
	CP	0.848	0.884	0.920	0.940	0.766	0.836	0.920	0.942

*μ_x_* = 1.5, $\sigma_{x}^{2}$ = 1, $\sigma_{u}^{2}$ = 1, $\overset{¯}{\eta }$ = 86%									
	
*β*_0_ = 0	Bias	0.175	−0.276	−0.014	−0.015	0.171	−0.277	−0.017	−0.016
	SD	0.186	0.277	0.222	0.230	0.135	0.203	0.166	0.172
	ASE	0.177	0.267	0.214	0.223	0.125	0.188	0.150	0.156
	CP	0.824	0.800	0.938	0.948	0.700	0.672	0.934	0.940
*β*_1_ = ln(2)	Bias	−0.173	0.108	−0.014	0.011	−0.171	0.109	−0.012	0.012
	SD	0.113	0.178	0.146	0.162	0.081	0.128	0.106	0.117
	ASE	0.108	0.171	0.140	0.155	0.076	0.121	0.098	0.109
	CP	0.610	0.914	0.948	0.946	0.404	0.856	0.926	0.940

*β*_0_ = 0	Bias	0.232	−0.487	−0.028	−0.023	0.225	−0.487	−0.031	−0.023
	SD	0.204	0.333	0.249	0.269	0.146	0.236	0.183	0.199
	ASE	0.193	0.314	0.238	0.259	0.136	0.221	0.167	0.181
	CP	0.754	0.642	0.946	0.952	0.626	0.398	0.924	0.922
*β*_1_ = ln(3)	Bias	−0.273	0.175	−0.056	0.023	−0.270	0.174	−0.055	0.021
	SD	0.148	0.240	0.183	0.227	0.104	0.166	0.129	0.162
	ASE	0.138	0.222	0.171	0.213	0.098	0.156	0.120	0.148
	CP	0.488	0.892	0.900	0.946	0.230	0.824	0.902	0.940

NOTE: See the footnote of Table 1 for notation.

**Table 4.** Simulation study for linear regression model with truncated surrogates; covariates are *X* and *Z*.

		Naive	RC	CRC	EEE	Naive	RC	CRC	EEE

		*n* = 500				*n* = 1000			

*μ_x_* = 1.5, *σ_x_* = 1, *σ_u_* = 0.707, $\overset{¯}{\eta }$ = 89%									
	
*β*_0_ = 0.5	Bias	0.137	−0.225	−0.006	−0.001	0.134	−0.224	−0.001	0.005
	SD	0.095	0.122	0.109	0.110	0.065	0.082	0.074	0.073
	ASE	0.093	0.117	0.106	0.106	0.066	0.083	0.075	0.074
	CP	0.694	0.504	0.938	0.930	0.454	0.226	0.946	0.944
*β*_1_ = 1	Bias	−0.137	0.094	0.004	0.001	−0.136	0.093	0.001	−0.003
	SD	0.051	0.071	0.071	0.065	0.033	0.048	0.044	0.043
	ASE	0.050	0.069	0.064	0.063	0.036	0.049	0.044	0.044
	CP	0.204	0.742	0.940	0.938	0.020	0.538	0.954	0.956
*β*_2_ = −1	Bias	0.042	0.042	−0.004	−0.004	0.049	0.049	0.002	0.003
	SD	0.052	0.052	0.053	0.053	0.036	0.036	0.038	0.037
	ASE	0.050	0.050	0.050	0.050	0.035	0.035	0.036	0.036
	CP	0.852	0.852	0.938	0.938	0.704	0.704	0.942	0.942
	
*μ_x_* = 1.5, *σ_x_* = 1, *σ_u_* = 1, $\overset{¯}{\eta }$ = 86%									
	
*β*_0_ = 0.5	Bias	0.300	−0.347	−0.016	−0.016	0.298	−0.338	−0.005	−0.004
	SD	0.098	0.170	0.142	0.143	0.067	0.114	0.095	0.094
	ASE	0.095	0.162	0.136	0.136	0.067	0.113	0.095	0.094
	CP	0.110	0.406	0.944	0.944	0.006	0.132	0.956	0.954
*β*_1_ = 1	Bias	−0.264	0.138	0.011	0.011	−0.264	0.132	0.004	0.002
	SD	0.051	0.099	0.087	0.087	0.033	0.068	0.060	0.059
	ASE	0.049	0.096	0.083	0.083	0.035	0.068	0.058	0.058
	CP	0.000	0.732	0.944	0.948	0.000	0.518	0.958	0.958
*β*_2_ = −1	Bias	0.070	0.070	−0.005	−0.006	0.076	0.076	0.002	0.002
	SD	0.054	0.054	0.059	0.059	0.038	0.038	0.042	0.042
	ASE	0.052	0.052	0.053	0.054	0.037	0.037	0.038	0.038
	CP	0.736	0.736	0.934	0.938	0.464	0.464	0.922	0.920

NOTE: Naive is an estimator that uses the average of two replicates as the covariate, RC is the usual RC estimator that uses $\text{E}(X|\tilde{W},Z)$ as the covariate, CRC is a conditional RC estimator that uses $\text{E}(X|\tilde{W},Z,\eta )$ as the covariate, EEE is the expected estimating equation estimator described.

**Table 5.** Analysis results of data from the APPEAL study.

		Naive	NRC	RC	EEE
	
Intercept	*β*_0_	0.259	0.345	0.299	0.282
	SE	0.360	0.377	0.367	0.364
log(MET+1)	*β*_1_	−0.067	−0.136	−0.107	−0.098
	SE	0.045	0.098	0.071	0.062
Age	*β*_2_	0.015	0.015	0.014	0.015
	SE	0.006	0.006	0.007	0.007

		Nuisance parameters			
	*μ_x_*		1.258	0.925	0.927
	SE		0.100	0.160	0.161
	$\sigma_{x}^{2}$		0.447	0.976	0.987
	SE		0.145	0.337	0.330
	$\sigma_{u}^{2}$		0.910	1.674	1.671
	SE		0.130	0.293	0.292

Note: See the footnote of Table 1 for notation. The percentages of non-zero log(1+MET) were 66.7% and 67.8% at baseline and 12 months among the participants in the control group, respectively. The total sample size in the analysis was 176.

## Data Availability

The data that support the findings of this study are not available for public access at this moment, but can be requested from the APPEAL study.

## Appendix A. Proofs of Propositions 1 and 2

### Proof of Proposition 1.

Based on a standard surrogate assumption, the measurement errors $U_{ij}$ and $e_{i}$ are independent. Also, the truncation indicator ${\tilde{\eta }}_{i}$ is independent of $e_{i}$. Hence, $E\left(e_{i}|{\tilde{W}}_{i},Z_{i}\right)=0$. The unbiasedness of the estimating Equation (2) of the RC estimator can be obtained by calculating the expectation of the estimating score for individual i,

$$\begin{array}{ll} & E\left[{\left(1,{\hat{X}}_{i},Z_{i}^{'}\right)}^{'}\{Y_{i}-(\beta_{0}+\beta_{1}{\hat{X}}_{i}+\beta_{2}^{'}Z_{i})\}\right.\\ =E({\left(1,{\hat{X}}_{i},Z_{i}^{'}\right)}^{'}E[\{Y_{i}-(\beta_{0}+\beta_{1}{\hat{X}}_{i}+\beta_{2}^{'}Z_{i})\}|{\tilde{W}}_{i},Z_{i}])\\ =0. \end{array}$$

Hence, for linear regression with zero-inflated surrogates, the RC estimator is consistent.

### Proof of Proposition 2.

We note that $\phi \left(Y_{i},X_{i},Z_{i},\beta \right)$ is an estimating score that satisfies $E\left\{\phi \left(Y_{i},X_{i},Z_{i},\beta \right)\right\}=0$. We note that

$$E\left[E\left\{\phi \left(Y_{i},X_{i},Z_{i},\beta \right)|Y_{i},{\tilde{W}}_{i},Z_{i}\right\}\right]=E\left\{\phi \left(Y_{i},X_{i},Z_{i},\beta \right)\right\}=0.$$

Hence, estimating Equation (4) for the EEE estimator is unbiased. We now develop the asymptotic distribution of the EEE estimator. Let the estimating score of the EEE estimator for the ith participant $E\left\{\phi \left(Y_{i},X_{i},Z_{i},\beta \right)|Y_{i},{\tilde{W}}_{i},Z_{i}\right\}$ be denoted by $\psi \left(Y_{i},{\tilde{W}}_{i},Z_{i},\beta \right)$. Let $G(\beta )=-E\{\partial \psi (Y,\tilde{W},Z,\beta )/\partial \beta \}$. By a Taylor expansion of the estimating equation at the true β, and under some regularity conditions, it can be shown that

$$n^{1/2}({\hat{\beta }}_{eee}-\beta )=G^{-1}\left(\beta \right)n^{-1/2}\sum_{i=1}^{n}\psi (Y_{i},{\tilde{W}}_{i},Z_{i},\beta )+o_{p}\left(1\right),$$

Hence, it is seen that $n^{1/2}({\hat{\beta }}_{\mathrm{eee}}-\beta )$ is asymptotically normal with mean 0 and variance

$$\{G\left(\beta \right){\}}^{-1}n^{-1}[\sum_{i=1}^{n}\;\psi (Y_{i},{\tilde{W}}_{i},Z_{i},\beta ){\left\{\psi \left(Y_{i},{\tilde{W}}_{i},Z_{i},\beta \right)\right\}}^{'}]{\left\{G^{-1}\left(\beta \right)\right\}}^{'},$$
